# Annotations

**Published:** 1899-12-02

**Authors:** 


### Annotations.

### Medical Attendance on the Families of Soldiers and Sailors on Active Service.

The excellent suggestion which seems to have cropped up almost simultaneously from various sources within the profession, to the effect that medical men should place their professional services at the disposal of the wives and children of all soldiers and sailors who have been called to the front must, we think, receive general and hearty approval. There is one point, however, which we think is worthy of consideration. In view of the enormous mass of gratuitous medical service which is given to all sorts of people by medical men, and often remains not only unacknowledged by, but unknown by the public, we should be sorry to see the very large and important gift which it is proposed that the medical profession should present, in kind, towards the funds which are being collected for the relief of the wives and children of our soldiers, thrown in with the rest of the gratuitous medical services for which we receive so little thanks. A letter in the *Times* from the Hon. Secretary of the Soldiers' and Sailors' Families' Association shows what a large sum the proposed subscription of the medical men in the various parts of the country will amount to, and while we in every way approve of the suggestion that medical men should give this contribution, we think that—acting on the general principle of not hiding one's light under a bushel, and of giving to each man the credit of his good deeds—some arrangement should be made, in accordance with which every medical man who contributes of his services to the relief of those with whom the country is just now in such full sympathy should be credited in the local subscription lists with the full value of the services so rendered. What we are quite clear about is that in many districts the contribution of the doctor will be taken as a matter of course, and thought but little of by many good people, who, on the strength of a guinea subscription, propose to run the show—people who would be vastly astonished if they were asked to subscribe to the funds the 10 or 20 guineas which, under this arrangement, many a medical man will be called upon to give.

### The Question of the Midwives Once Again.

A large gathering of medical men came together in Newcastle last week to hear addresses from the direct representatives of the profession in the General Medical Council, and to discuss the medical politics of the day. We have not space here to consider all the topics which were touched upon, but we must express our disappointment at the attitude taken up by the great majority of those present upon the midwives' question. What we have to face is the fact that we have always had the "female midwife," as she was called, with us, and that the only questions which will excite interest at Westminster will be how best to compel these hitherto ignorant women to become educated, and how to ensure that in their work they shall conform to a proper standard. As Mr. Horsley said plainly enough, members of Parliament know nothing about the privileges of the profession, but they intend to vote so that the calamities caused by ignorant midwives shall be stopped, and they see quite well that this can only be done by putting these women on a register. Clearly if, as a profession, we choose to stand on one side and wring our hands, legislation will take place over our heads without any reference to us. Yet we find men saying at Newcastle that they "object to any Bill and they object to every Bill," and that "the only straightforward course for practitioners to take is to say to the General Medical Council, to Parliament, and to the Privy Council that they may go on with their Midwives Bill, but that they, the practitioners, protest that it is not a good thing for the public or for the profession, and they will do their best to defeat it, and will wash their hands of it, and have none of it." This seems to us to be an absolutely unreasonable position to take up. If the medical profession possesses any such political influence as to make it in the slightest degree probable that by washing its hands of the matter it can interfere with legislation on the subject, then such influence should certainly be put to better purpose than that of merely obstructing "any and every" Bill that is brought in to remove what is admitted to be a scandal and a serious danger to parturient women. We should guide legislation rather than impede it; for, indeed, legislation sadly wants guiding in this matter. As Mr. Victor Horsley said, "the extirpation of every midwife in the country is an impossible and absurd suggestion," yet "it is the duty of the profession to see that these midwifery catastrophes are stopped." Some kind of regulation of midwives is clearly necessary. But these Northerners are a stubborn race, and they went their way, the motion being carried with one dissentient. Whether, however, that meant that the meeting agreed with the words of the resolution or with the speeches of its proposers there was nothing to show.

### Red Noses.

A Berlin medical paper points out the influence of the veil, so commonly worn by women in winter, in producing redness of the nose. This effect is said to be due partly to the mechanical action of the rough texture upon the delicate skin, and partly to the influence of the watery vapour which collects and condenses within its texture. It is suggested that the treatment consists in discarding the veil as a first measure; but in deference, we suppose, to the exigencies of fashion in regard to fluffy hair, it is recommended that when the use of a veil is necessary it should not extend lower than the orifices of the nose, so that the watery vapour from the breath should not be confined by it. We do not think that ladies will give up wearing veils even with bonnets, but perhaps the fear of red noses may encourage them in their preference for hats. Certainly a veil descending from the brim of a hat and tucked under the chin is a more reasonable arrangement than that of having one drawn tightly across the face and compressing the nose, as is so often done by the wearers of the things which are, we believe, still called bonnets. In fact, we are not altogether sure that a good deal might not be said in favour of a properly arranged veil, leaving plenty of air space around the face, even from a sanitary point of view. In the country it is, of course, another matter; but, considering the filth which must enter the mouth in the course of a walk in town upon a dusty day, the slight rebreathing of the air which the presence of the veil involves might be more than compensated for by the increased freedom from the grosser kinds of dirt which it should ensure

